# The ribosome assembly GTPase EngA is involved in redox signaling in cyanobacteria

**DOI:** 10.3389/fmicb.2023.1242616

**Published:** 2023-08-10

**Authors:** Antonio Llop, Sirine Bibak, Raquel Cantos, Paloma Salinas, Asunción Contreras

**Affiliations:** Departamento de Fisiología, Genética y Microbiología, Facultad de Ciencias, Universidad de Alicante, Alicante, Spain

**Keywords:** cold acclimatization, PipX, high light acclimatization, PII, *Synechococcus elongatus*, phenotypic analysis, nitrogen regulation network, translation

## Abstract

Photosynthetic organisms must cope with environmental challenges, like those imposed by the succession of days and nights or by sudden changes in light intensities, that trigger global changes in gene expression and metabolism. The photosynthesis machinery is particularly susceptible to environmental changes and adaptation to them often involves redox-sensing proteins that are the targets of reactive oxygen species generated by photosynthesis activity. Here we show that EngA, an essential GTPase and ribosome-assembly protein involved in ribosome biogenesis in bacteria and chloroplasts, also plays a role in acclimatization to environmentally relevant stress in *Synechococcus elongatus* PCC7942 and that PipX, a promiscuous regulatory protein that binds to EngA, appears to fine-tune EngA activity. During growth in cold or high light conditions, the EngA levels rise, with a concomitant increase of the EngA/PipX ratio. However, a sudden increase in light intensity turns EngA into a growth inhibitor, a response involving residue Cys122 of EngA, which is part of the GD1-G4 motif NKCES of EngA proteins, with the cysteine conserved just in the cyanobacteria-chloroplast lineage. This work expands the repertoire of ribosome-related factors transmitting redox signals in photosynthetic organisms and provides additional insights into the complexity of the regulatory interactions mediated by EngA and PipX.

## Introduction

EngA (YphC/Der/YfgK), a ribosome-assembly protein conserved in bacteria, belongs to the GTPase superfamily (Hwang and Inouye, 2006; Bharat and Brown, 2014) and is involved in ribosome biogenesis in bacteria and chloroplasts. The closest homologs of the plant chloroplast proteins (EngA1) are the cyanobacterial EngA proteins (Suwastika et al., 2014). So far, the *engA* gene has proven essential in all systems studied (Hwang and Inouye, 2006; Bharat and Brown, 2014; Jeon et al., 2014; Ni et al., 2016; Jerez et al., 2021). Several phenotypes have been associated with the depletion or overexpression of EngA. Depletion phenotypes include cold sensitivity, cell filamentation, abnormal cell curvature, apparent nucleoid condensation, and in plants, aberrant thylakoid organization (Hwang and Inouye, 2001; Morimoto et al., 2002; Bharat and Brown, 2014; Jeon et al., 2014; Jerez et al., 2021). EngA overexpression phenotypes include changes in cell wall structure and morphology in *Escherichia coli* (Lee et al., 2011) and leaf variegation, high chlorophyll fluorescence, increased ROS generation, and compromised PSII repair in *Arabidopsis thaliana* (Kato et al., 2018). However, in the cyanobacterium *Synechococcus elongatus* PCC7942 (hereafter *S. elongatus*) overexpression of EngA had no negative effect on growth or phenotypic impact under standard growth conditions (Jerez et al., 2021).

EngA is composed of two GTPase domains (GD1 and GD2) in tandem and a C-terminal domain resembling in its architecture a KH fold (RNA-binding module; Robinson et al., 2002; Hwang and Inouye, 2006; Valverde et al., 2008; Verstraeten et al., 2011). In each of the GD domains, four characteristic sequence motifs (G1, G2, G3, and G4; Bourne et al., 1991), are involved in binding GTP or GDP, that in turn affect interdomain interactions that ultimately regulate EngA activity (Majumdar et al., 2017). Several structures of EngA corresponding to different conformations of this protein have been reported and analyzed (Robinson et al., 2002; Foucher et al., 2012; Zhang et al., 2014; Majumdar et al., 2017; Upendra and Krishnaveni, 2020), but none of these structures corresponds to EngA from cyanobacteria or chloroplasts.

EngA has recently been identified as a component of the PII interacting protein X (PipX) synteny and interaction networks in *S. elongatus* (Labella et al., 2020a; Jerez et al., 2021). PipX, which interacts with the GD1 domain of EngA in a guanosine diphosphate-dependent manner *in vitro* (Jerez et al., 2021) is a small protein that binds to the widely distributed and highly conserved signal transduction protein PII (Burillo et al., 2004; Espinosa et al., 2006, 2009, 2010; Laichoubi et al., 2012) encoded by *glnB*, and to the cyanobacterial global transcriptional regulator NtcA (Espinosa et al., 2006, 2010; Llácer et al., 2010; Laichoubi et al., 2012). Both PII and NtcA are sensors of 2-oxoglutarate (2-OG), a universal indicator of the intracellular carbon-to-nitrogen balance (Huergo and Dixon, 2015). PII also senses ATP/ADP and thus PipX swapping between PII and NtcA links PII signaling of carbon-to-nitrogen balance and energy with NtcA-regulated gene expression (Espinosa et al., 2006, 2007, 2014, 2018, Llácer et al., 2010, Zeth et al., 2014, Forchhammer and Selim, 2020, Labella et al., 2020b).

PII and PipX mediate protein–protein interactions with multiple targets, constituting the hubs of a complex and dynamic interaction network for metabolic homeostasis in cyanobacteria (Labella et al., 2017, 2020b; Tremiño et al., 2022). Targets of PII regulation include transcriptional regulators, enzymes, and transporters involved in nitrogen and carbon assimilation (Burillo et al., 2004; Llácer et al., 2007, 2008; Forcada-Nadal et al., 2018; Labella et al., 2020b; Forchhammer et al., 2022). Cyanobacterial genomes always contain at least as many copies of *glnB* as of *pipX* (Laichoubi et al., 2011) and in *S. elongatus*, a high PipX/PII ratio prevents growth (Espinosa et al., 2009, 2010; Laichoubi et al., 2012; Chang et al., 2013; Jerez et al., 2021; Llop et al., 2023) suggesting that a relatively high ratio of PII over PipX is required to counteract unwanted interactions with less abundant and/or low affinity PipX partners. So far, EngA is the best candidate for this predicted PipX binding protein.

During cold stress, PipX and EngA have opposite (negative and positive, respectively) effects on growth (Jerez et al., 2021), suggesting that PipX binds to EngA to interfere with ribosome assembly and slow down growth under conditions in which a significant number of PipX-EngA complexes form.

**Figure 1:**
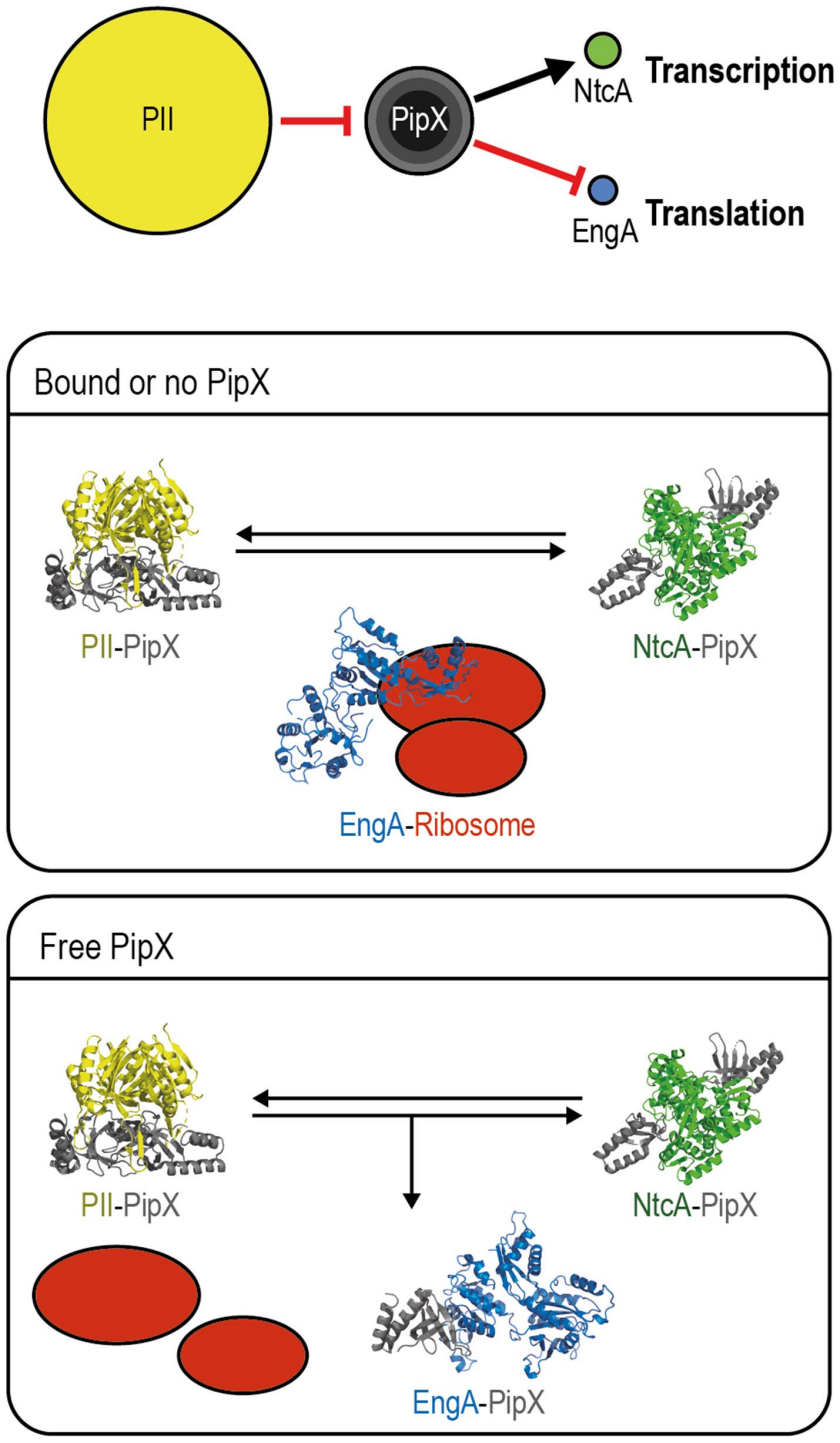
Proposed model for PipX regulation of EngA. Top panel: Schematic representation of the signaling pathway from PII to NtcA and EngA with indication of the relevant protein–protein interactions and alternative scenarios for PipX-EngA interactions. Circle areas are proportional to protein abundance (PII trimers; PipX: trimers, dimers and monomers from inner to outer circles, respectively; NtcA dimers; and EngA monomers). The red hammer head lines and the black arrow indicate inactivation by sequestration and co-activation, respectively. Middle panel “Bound or no PipX”: under standard culture conditions PipX is involved in partner swapping with PII and NtcA while EngA is active. Bottom panel “Free PipX”: when the levels of free PipX raise, it also binds to EngA, interfering with its ribosome-assembly function. PDB models: PII-PipX from 2XG8; NtcA-PipX from 2XKO; closed EngA predicted with SWISS model using as query *S. elongatus* protein sequence based on the PDB 4DCS model; open EngA from *Escherichia coli* model 3J8G. Re-published from Jerez et al. (2021).

The current model for PipX regulation of EngA activity, summarized in Figure 1 considers that (a) PipX-EngA complex formation would depend on the levels of PII and EngA effectors, together signaling energy and carbon-to-nitrogen balance, and (b) in *S. elongatus* cultures growing under standard laboratory conditions PII is in 14-fold excess over PipX, which is in a roughly similar excess over its regulatory targets NtcA and EngA (Guerreiro et al., 2014; Labella et al., 2016). However, it is not known whether the EngA/PipX ratio changes under environmentally relevant conditions such as cold stress, where PipX counteracts EngA activity.

The aim of this work was to gain additional insights into the functions and peculiarities of cyanobacterial EngA and its regulatory connections with PipX. We have shown that upregulation of EngA levels, with a concomitant increase of the EngA/PipX ratio, takes place during acclimatization to environmentally relevant conditions such as low temperature or high light. *In silico* analysis of EngA revealed distinctive structural features conserved in the cyanobacteria-chloroplast lineage. One of these cyanobacterial signatures is the presence of a conserved cysteine at the GD1-G4 motif (NKXES in bacteria) of cyanobacterial EngA proteins, which mediates a growth inhibition response triggered by high light in *S. elongatus*. This work expands the repertoire of ribosome-related factors transmitting redox signals in photosynthetic organisms and provides additional insights into the complexity of the regulatory interactions mediated by EngA and PipX.

## Materials and methods

### Cyanobacteria culture conditions and growth assays

Cultures were routinely grown in BG11 media [BG11_0_ plus 17.5 mM sodium nitrate (NaNO_3_) and 10 mM HEPES/NaOH pH 7.8; Rippka et al., 1979] photo-autotrophically under constant illumination provided by cool white fluorescent lights at 30°C in baffled flasks (shaking: 150 rpm, 70 μmol photons m^−2^ s^−1^) or on plates (50 μmol photons m^−2^ s^−1^). For solid media, 1.5% (*w*/*v*) agar and, after autoclaving, 0.5 mM sodium thiosulfate (Na_2_S_2_O_3_) were added. To select genetically modified strains the antibiotics chloramphenicol (Cm; 3.5 μg mL^−1^), streptomycin (Sm; 15 μg mL^−1^), or nourseothricin (Nt; 15 μg mL^−1^) were used. The transformation was performed essentially as described by Golden and Sherman (1984).

To monitor the growth, optical density at 750 nm (OD_750nm_) was measured using a Ultrospec 2,100 pro UV–Vis Spectrophotometer (Amersham). For growth in liquid, 30–50 mL of cultures were adjusted to an initial OD_750nm_ of 0.1 and grown until they reach 0.5–0.6 (timepoint 0). Cold conditions (18°C) were performed in a FOC 200I Connect Cooled Incubator (VELP Scientifica) with cool white fluorescent lights (40 μmol photons m^−2^ s^−1^). Low (LL) and moderate light (ML) conditions (30°C and 2–3 or 70 μmol photons m^−2^ s^−1^, respectively) were provided by cool white fluorescent lights, and high light conditions (HL, 1000 μmol photons m^−2^ s^−1^) by a MASTER SON-T PIA Plus 250 W E40 lamp.

For growth on solid media, exponentially growing cultures (mix of approximately the same amount of biomess from three clones) were adjusted to 0.5 before dropping 5 μL of the cell suspensions and serial dilutions (5^−1^, 10^−1^, and 10^−2^) onto BG11 plates. Isopropyl β-D-1-thiogalactopyranoside (IPTG) was added as indicated. Cells were grown at 30°C under standard conditions, high light intensity (400 μmol photons m^−2^ s^−1^, provided by cool white LED lights), or exposed to a very high light pulse (VHL, 850 μmol photons m^−2^ s^−1^, provided by a MASTER SON-T PIA Plus 250 W E40 lamp) for 20 min and then grown under standard illumination for 5 days. To induce EngA expression before a VHL pulse, 3^N^Ptrc-EngA and 3^N^Ptrc^Osym^-EngA/*ΔengA* cultures were grown in liquid BG11 for 2 h with the addition of 1 mM IPTG, washed with BG11, and adjusted to OD_750nm_ 0.5 before doing the drops in BG11 plates, grown at moderate light. To maximize the differences, photographs were always taken after 5 days growing using a Nikon camera at the default parameters. Pictures were analyzed using ImageJ. Circular regions of interest (ROI) were manually generated for each drop, and the average pixel intensity in the red channel was retrieved. An empty ROI was generated to obtain the background noise value of each picture which was subtracted from the other ROI average values. The growth of each strain relative to the control strain was calculated as the minimum value of the ratios between the measured biomass values of drops of the same dilution. Wilcoxon rank sum test with Bonferroni correction versus the control strain was performed with the RStudio program (RStudio Team, 2020).

### Plasmid and strains construction

All plasmids constructed in this work (Table 1) were analyzed by automated dideoxy DNA sequencing. Primers used are described in Supplementary Table S1.

**Table 1:** Plasmids used in this work.

**Plasmids**	**Genotype or relevant characteristics**	**Source or references**
pUAGC70	NS3, *Ptrc lacI*, Nt^R^	Espinosa et al., 2018
pUAGC77	NS3, *Ptrc::engA lacI*, Nt^R^	Jerez et al., 2021
pUAGC87	NS3, *Ptrc^Osym^::engA lacI*, Nt^R^	This work
pUAGC37	NS3, *Ptrc^Osym^::engA^364tgc>gct^ lacI*, Nt^R^	This work
pUAGC38	NS3, *Ptrc^Osym^::engA^365g>c^ lacI*, Nt^R^	This work
pUAGC873	NS1*, Ptrc::pipX lacI*, Ap^R^ Sm^R^	Labella et al., 2017
pUAGC126	*pipX* replaced with *cat*, Ap^R^ Cm^R^	Labella et al., 2017
pUAGC908	*engA* replaced with *cat*, Ap^R^ Cm^R^	Jerez et al., 2021

Ap, ampicillin, Sm, streptomycin, Cm, chloramphenicol, Nt, nourseothricin.

R , resistance *cat*, chloramphenicol acetyltransferase.

To obtain pUAGC87, plasmid pUAGC77 was mutated with the primer pair Ptrc_symmetric_F/Ptrc_symmetric_R. To obtain pUAGC37 and pUAGC38, plasmid pUAGC87 was used for mutagenic PCR with primers Mut_C122A-F/Mut_C122A-R and EngA-C122S-For/EngA-C122S-Rev, respectively.

To obtain strains 3^N^Ptrc, 3^N^Ptrc-EngA, 3^N^Ptrc^Osym^-EngA, 3^N^Ptrc^Osym^-EngA^C122A^, 3^N^Ptrc^Osym^-EngA^C122S^, 1^S^Ptrc-PipX, and *ΔpipX* (Table 2), wild-type *S. elongatus* was transformed with plasmids pUAGC70, pUAGC77, pUAGC87, pUAGC37, pUAGC38, pUAGC873 or pUAGC126, respectively. Verification of the correct insertion at the NS3 neutral site or of inactivation of *pipX* was confirmed by PCR analysis with oligonucleotide pairs NS3seq-1F/NS3seq-1R and PIPX-5R-129/PipX-126-F, respectively.

**Table 2:** Strains used in this work.

**Strains**	**Genotype or relevant characteristics**	**Source or references**	
WT	Wild type *S. elongatus* PCC7942	Pasteur Culture Collection	
*ΔpipX*	*pipX*::*cat*, Cm^R^	Labella et al., 2017	
3^N^Ptrc	Φ(NS3-*Ptrc*), Nt^R^	Espinosa et al., 2018	
3^N^Ptrc-EngA	Φ(NS3-*Ptrc::engA*), Nt^R^	Jerez et al., 2021	
3^N^Ptrc-EngA /*ΔengA*	Φ(NS3-*Ptrc::engA*) / *engA::cat*, Nt^R^ Cm^R^	Jerez et al., 2021	
3^N^Ptrc^Osym^-EngA	Φ(NS3-*Ptrc^Osym^::engA*), Nt^R^	This work	
3^N^Ptrc^Osym^-EngA/*ΔengA*	Φ(NS3- *Ptrc^Osym^::engA*) / *engA::cat*, Nt^R^ Cm^R^	This work
3^N^Ptrc^Osym^-EngA^C122A^	Φ(NS3- *Ptrc^Osym^*::*engA^364tgc>gct^*), Nt^R^	This work	
3^N^Ptrc^Osym^-EngA^C122A^/*ΔengA*	Φ(NS3- *Ptrc^Osym^*::*engA^364tgc>gct^*) / *engA::cat*, Nt^R^ Cm^R^	This work	
3^N^Ptrc^Osym^-EngA^C122S^	Φ(NS3- *Ptrc^Osym^*::*engA^365g>c^*), Nt^R^	This work	
3^N^Ptrc^Osym^-EngA^C122S^/*ΔengA*	Φ(NS3- *Ptrc^Osym^*::*engA^365g>c^*) / *engA::cat*, Nt^R^ Cm^R^	This work	
1^S^Ptrc-PipX	Φ (NSI-*Ptrc*::*pipX*), Sm^R^	Labella et al., 2017	

Ap, ampicillin, Sm, streptomycin, Cm, chloramphenicol, Nt, nourseothricin.

R , resistance *cat*, chloramphenicol acetyltransferase.

### Protein extraction, immunodetection, and band quantification

To perform immunodetection of PipX, EngA, and PII in *S. elongatus*, 10 mL of cultures from the growth curves were harvested by centrifugation (7,300 g for 6 min at 4°C) at different times. The pellets were lysed in 100 μL of lysis buffer (50 mM Tris/HCl pH 7.4, 4 mM EDTA, 0.5 mM PMSF, 0.5 mM benzamidine, 1 mM DTT) with the addition of 0.1 μm glass beads, following the procedure described in Labella et al. (2016). Cells were subjected to three cycles of 60 s disruption at a speed of 5 m/s in a high-speed homogenizer Minibeadbeater and 60 s of resting at 4°C. The lysates were centrifuged (5,500 g for 5 min at 4°C) and the supernatant fractions were extracted, collected, and quantified by the Bradford method using a detergent compatible Bradford assay kit (Pierce^™^) in a VICTOR3^™^ 1,420 Multilabel Plate Reader (PerkinElmer). The protein extracts were stored at − 20°C until needed.

To separate the proteins, the extracts were charged in sodium dodecyl sulfate polyacrylamide gel electrophoresis (SDS-PAGE; 10–20% polyacrylamide linear gradient) using two independent gels (one for detecting EngA and PII and the other for PipX and PII), followed by immunoblotting in 0.1-μm polyvinylidene fluoride membranes (from GE Healthcare), maintaining the temperature at 4°C. The membranes were blocked with Tris-Buffered Saline (TBS; 20 mM Tris/HCl pH 7.5, 500 mM NaCl) solution containing 5% nonfat dried milk for 30 min at room temperature and then incubated overnight in TBS solution containing 2% nonfat dried milk and the primary antibody (diluted at 1:5000 for PipX and EngA, and 1:10000 for PII). Rabbit antisera against EngA were obtained from Pineda Antikörper Service (Berlin, Germany) using ∼ 2 mg of pure recombinant His-EngA as an antigen and following a 60-day immunization protocol. The antiserum against PipX and PII proteins was donated by K. Forchhammer (Univ. Tübingen, Germany). The membranes were then incubated at room temperature for 1.5 h with a 1:150,000 dilution of ECL rabbit IgG, HRP-linked F (ab′)2 fragment (from donkey; GE Healthcare). The signal was detected adding the SuperSignal WestFemto reagent (Pierce) and recorded in a Biorad ChemiDoc Imager using the automatic exposure mode, avoiding pixel saturation.

Protein band intensities were quantified using the ImageJ software, with the “*rectangle*” function and the “*wand*” tool used to measure the area plot corresponding to the signal intensity. The area from the corresponding immunodetection (EngA or PipX) was normalized using the area of PII or the area of an inner band from the corresponding gel, as indicated. Data were represented as ratios of stress/standard, mutant/WT alleles or directly as signal intensity, always referred to the timepoint 0 h. Wilcoxon rank sum test with Bonferroni correction versus the control strain was performed with the RStudio program (RStudio Team, 2020).

### Computational methods

EngA homologous sequences, labeled as COG1160 proteins, were obtained from the EGGNOG database v5.0. Sequences were split into cyanobacterial or non-cyanobacterial sequences according to their NCBI ID and the resulting sets of protein sequences were used as queries for MEME searches. MEME discriminative mode (Bailey and Elkan, 1994) was applied with two different criteria, both using the default parameters but reducing the maximum motif length to 12 or 18 residues. Cyanobacterial and non-cyanobacterial sequences were used as primary and control input sequences, respectively. Weblogos were generated using the raw aligned version of the sequences from EGGNOG.^1^

EngA homologous sequences in the *Streptophyta* phylum were also retrieved from the EGGNOG database v5.0 using *Arabidopsis thaliana* homolog as query (AT3G12080). Raw alignment sequences^2^ were realigned together with *S. elongatus* sequences using ClustalW with default parameters.

In all cases, to simplify the visualization of the weblogos, the alignments were trimmed for all positions in which *Synpcc7942_2340* presented a gap and then the weblogo3 online tool^3^ was used to generate the image using the color scheme “Chemistry.”

## Results and discussion

### EngA accumulates during cold stress in *S. elongatus*

Despite the essential involvement of EngA in ribosome biogenesis, a crucial and highly regulated process in all living cells, little is known about the control of EngA expression in model bacterial systems and there are no reports on the regulation of EngA protein levels at low temperature, conditions in which translation rates substantially decrease (Farewell and Neidhardt, 1998; Gottesman, 2018; Zhang et al., 2018; Cheng-Guang and Gualerzi, 2021). Cold sensitivity, presumably related to the temperature dependence of rRNA folding and the role of EngA as an rRNA chaperone, has been reported in Der-depleted strains of *Escherichia coli* (Bharat and Brown, 2014) and in *S. elongatus*. In this cyanobacterium, EngA has a stimulatory role on growth rate at low temperatures that is counteracted by PipX while overexpression of EngA has no impact during growth in standard conditions (Jerez et al., 2021).

**Figure 2:**
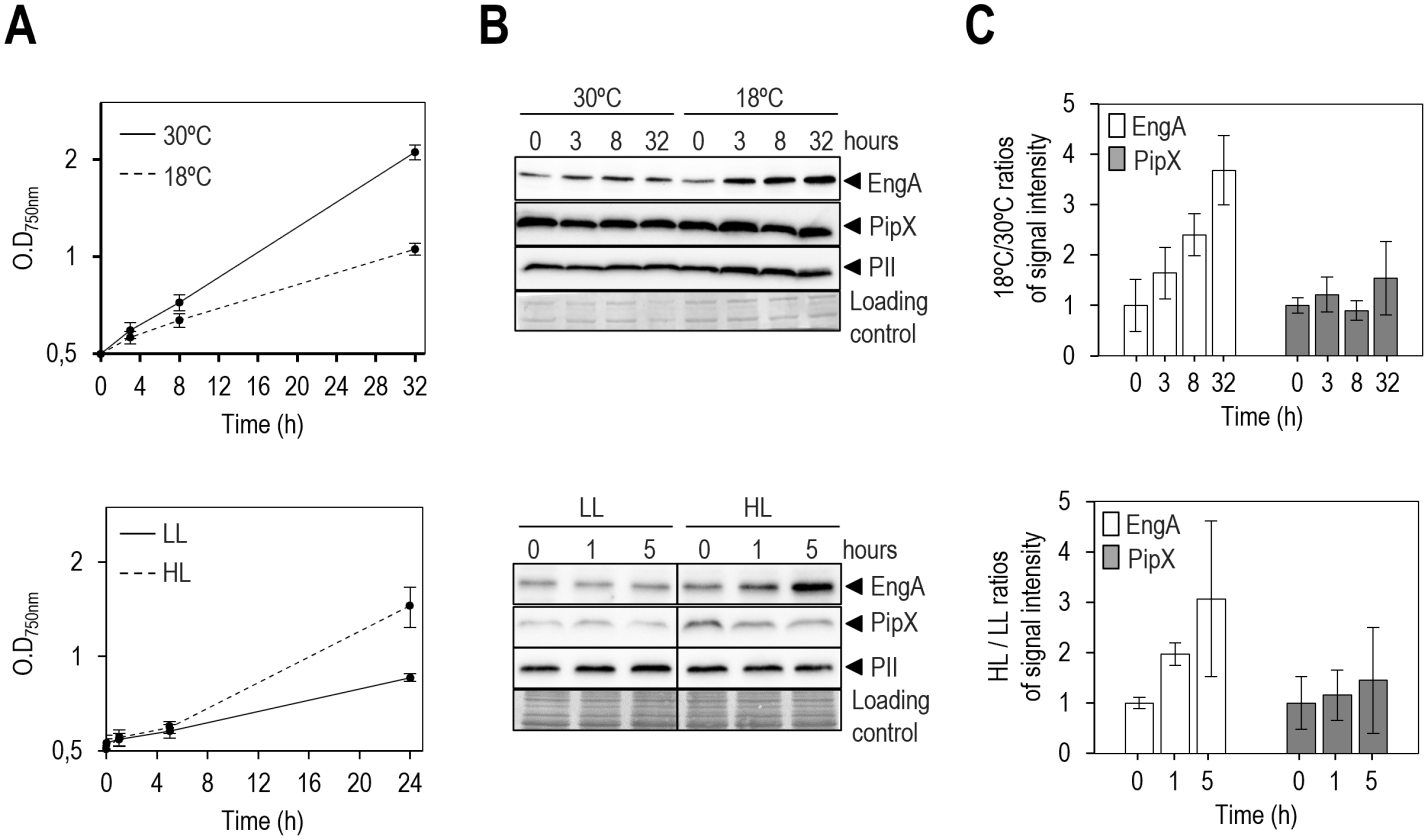
EngA levels under cold and high light stress in *S. elongatus*. **(A)** Growth curves (OD_750nm_) of *S. elongatus* cultures grown in parallel at standard (30°C) and low temperature conditions (18°C) or at low light (LL, 2–3 μmol m^−2^ s^−1^) and high light (HL, 1000 μmol m^−2^ s^−1^). **(B)** Representative immunodetection of PipX, EngA and PII proteins and fast green staining as a loading control. **(C)** Relative levels of the indicated proteins, previously normalized by PII and referred to the timepoint 0 h, shown as ratios of the values for the indicated culture conditions. The timepoint 0 always correspond to cultures that were growing at 30°C or at LL and then transferred to the same or different conditions, as indicated. Data are presented as means and error bars (standard deviation) of three biological replicates.

To determine whether EngA and/or PipX levels are affected by growth at low temperatures, we grew *S. elongatus* cultures at standard conditions before splitting them into two cultures to be grown at standard (30°C) or at low (18°C) temperature, two conditions in which growth rates are clearly different (Figure 2A). Subsequent immunodetection analysis of EngA, PipX and PII proteins revealed that while at 30°C all three protein levels remained constant during the length of the experiment, at 18°C the levels of EngA progressively increased (Figure 2B). To quantify the changes, we used the immunodetection signal for PII, indistinguishable amongst the different samples, as an internal control to normalize and calculate the 18°C/30°C ratios of the signal intensity for EngA or PipX bands. As shown in Figure 2C, there was a constant increase of EngA levels after transfer to 18°C, already apparent at the 3 h timepoint, and reaching almost 4-fold at the end of the 32 h long experiment. In clear contrast, PipX levels were independent of the temperature.

Temperature is a key environmental parameter, with low temperatures having major effects on biomass productivity and growth rate. The increase of EngA levels in response to cold stress supports the involvement of this ribosome-assembly factor in acclimatization to low temperatures, raising questions on whether it is a widespread phenomenon across bacteria or just a cyanobacterial peculiarity.

The increase of the EngA/PipX ratio provides additional parallelism between NtcA-PipX and EngA-PipX interactions in the context of transcription and translation regulation during nitrogen deficiency or cold stress, respectively. Since NtcA levels increase during nitrogen starvation (Espinosa et al., 2014), both PipX partners are upregulated under environmental conditions in which they are most required and this *per se* may increase the levels of the corresponding PipX complexes. However, while we know that 2-OG and high ATP/ADP are required to maximize NtcA-PipX complex formation, the molecular details governing EngA-PipX interactions during cold stress remain to be elucidated.

### EngA also accumulates during high light stress in *S. elongatus*

Light is the most relevant environmental parameter for photosynthetic organisms. It has a major impact on biomass productivity and growth rate, but excess light also damages the photosynthesis machinery and can inhibit growth. Therefore, acclimatization to strong light is a complex process requiring the implementation of a variety of protective mechanisms to reduce photoinhibition (Bailey and Grossman, 2008; Keren and Krieger-Liszkay, 2011; Muramatsu and Hihara, 2012). *S. elongatus* cultures that are acclimated to high light often show the yellowish appearance typical of stressed cultures undergoing chlorosis (Llop et al., 2023) but can nevertheless grow as fast or even faster than at the moderate light intensities used as standard conditions.

To gain further insights into the environmental regulation of EngA and a possible correlation between EngA levels and growth rates, we next compared the levels of EngA in *S. elongatus* cultures growing under two very different light intensities, defined here as low (LL, 2–3 μmol m^−2^ s^−1^) or high light (HL, 1000 μmol m^−2^ s^−1^) conditions. As shown in Figure 2A, these non-standard culture conditions resulted in slower (LL curve) or a similar growth rate (HL curve) than control cultures (30° curve) at the moderate (standard) light conditions (70 μmol m^−2^ s^−1^) used in our laboratory.

Western analyses were carried out for 5 h following the transfer to high light, after which, due to important protein degradation, quantification became increasingly difficult from the chlorotic high light cultures. The results showed that EngA levels increased in extracts from cultures transferred to high light, increasing 3-fold just after 5 h (Figures 2B,C). Therefore, the results suggested that the enlargement of the EngA pool correlates with acclimatization to high light.

While the temperature dependence of rRNA folding explains the importance of an enlarged EngA pool during growth at low temperatures, it is intriguing that increases in the EngA pool also occur under high light, an environmental condition that should not compromise rRNA folding.

### Promoter sequences upstream *engA* are required for environmental regulation

**Figure 3:**
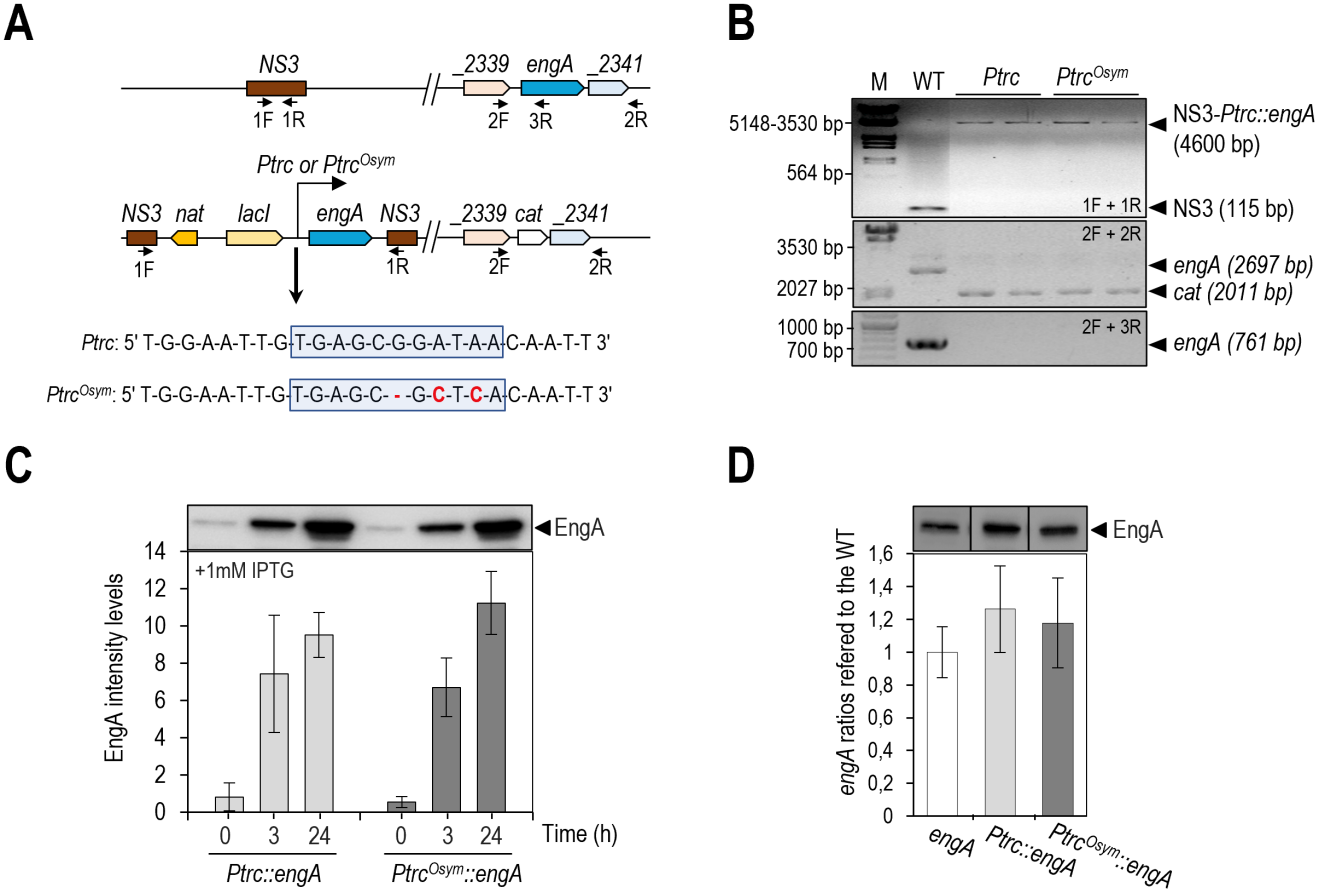
*Ptrc^Osym^*, a promoter with slightly less escape than *Ptrc*. **(A)** Schematic representation of relevant regions at the *S. elongatus* chromosome and of *Ptrc* and *Ptrc^Osym^* promoters. The symmetry of the operator is shown by blue boxes, the changed nucleotides are in red. **(B)** PCR results to verify the segregation at the NS3 region and *engA* inactivation in 3^N^Ptrc-EngA and 3^N^Ptrc^Osym^-EngA strains (abbreviated *Ptrc* and *Ptrc^Osym^*). **(C)** IPTG induction of EngA levels in *S. elongatus* strains differing in the indicated version of the ectopic allele at NS3. Data are presented as means and error bars (standard deviation) of three biological replicates. **(D)** EngA levels of strains expressing EngA only from the indicated allele. Three biological replicates from three independent experiments. Other details as in Figure 2.

To investigate the level at which upregulation of EngA levels takes place we next determined EngA levels in strain 3^N^Ptrc-EngA*/*Δ*engA* (see Figures 3A,B for details on strain construction), in which the ectopic *engA* gene is expressed from the Ptrc promoter while *cat* coding sequences, providing resistance to chloramphenicol-acetyltransferase, precisely replace those of *engA*. The corresponding allele (*Ptrc*::engA) is within a cassette encoding LacI repressor for control of gene expression and a selection marker (Nt^R^ for nourseothricin-resistance). To provide recombination sites at the *S. elongatus* chromosome for allelic replacement the cassette is flanked by sequences from the neutral site NS3 (see details in Figure 3A). Therefore in this strain the only source of EngA, the *Ptrc*::*engA* allele, contains just the *engA* coding sequences under the control of the Ptrc promoter.

**Figure 4:**
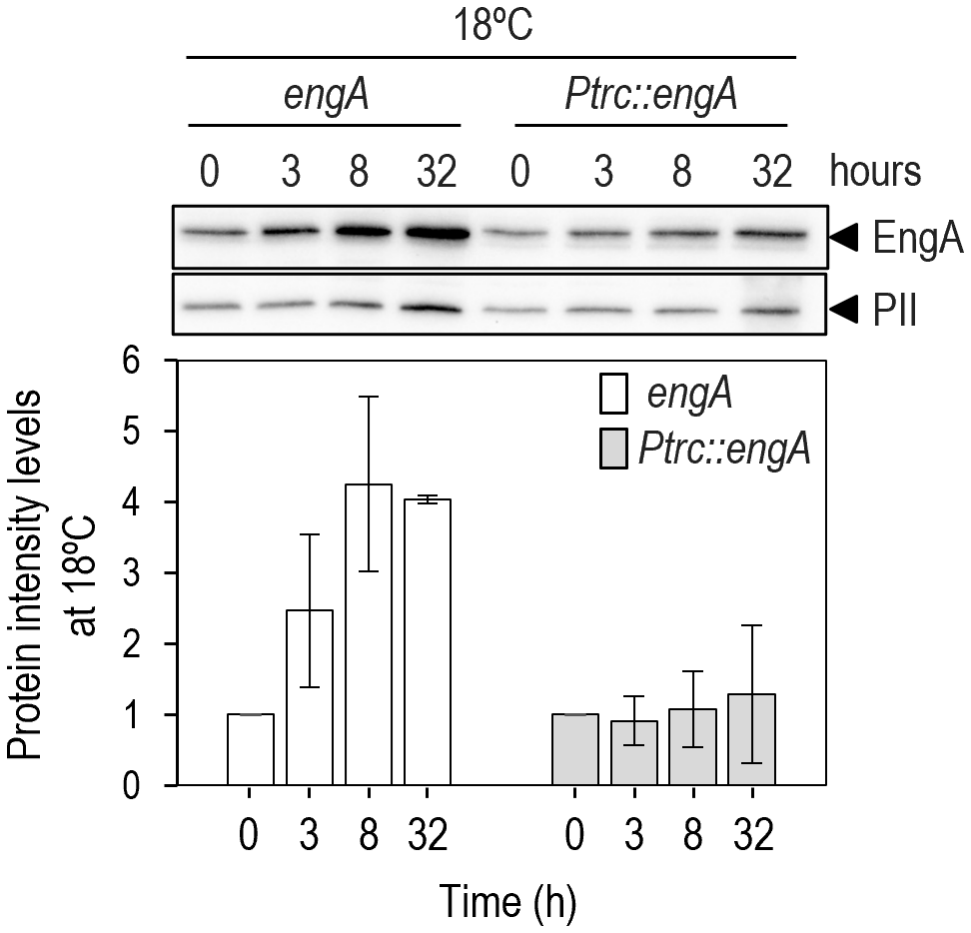
Promoter sequences upstream of *engA* are required for up regulation. Representative immunodetection of EngA and PII and relative protein levels from strains 3^N^Ptrc (*engA* allele) or 3^N^Ptrc-EngA/*ΔengA* (*Ptrc::engA*) transferred to 18°C. Protein intensity levels were normalized by PII and referred to the timepoint 0 h. Data are presented as means and error bars (standard deviation) of three biological replicates.

3^N^Ptrc-EngA*/*Δ*engA* and the wild-type control were analyzed in parallel. Cultures were grown at standard (30°C) conditions before splitting them into two cultures to be grown at the same (30°C) or at low (18°C) temperatures and subsequently analyzed by Western blots at different time points. As shown in Figure 4, *S. elongatus* cells expressing *engA* from the *Ptrc*::*engA* allele failed to increase EngA levels upon transfer to 18°C, in contrast to wild-type cells encoding the native *engA* locus. Therefore, *cis*-acting sequences upstream of the *engA* gene are required for upregulation in response to cold, suggesting that there is control at least at the transcriptional level.

### Engineering a *S. elongatus* strain with low and IPTG-inducible levels of EngA

Since *engA* is an essential gene that cannot be inactivated (Jerez et al., 2021), we attempted the construction of a conditional null strain in which the expression of *engA* was under the control of an efficiently repressed and inducible promoter, for which we tried to decrease the promoter leakage of the *Ptrc*::*engA* construct.

To increase the affinity of the LacI repressor for the *lac* operator controlling *Ptrc*::*engA* we introduced mutations to increase operator symmetry as described (Sadler et al., 1983 and Figure 3A). The resulting plasmid derivative (pUAGC87) and parental control (pUAGC77) were used to generate Nt^R^ transformants that were subsequently PCR-analyzed to verify homozygosis for the corresponding *Ptrc^Osym^*::*engA* and *Ptrc*::*engA* alleles (Figure 3B, top), generating strains 3^N^Ptrc^Osym^-EngA and 3^N^Ptrc-EngA, respectively. To confirm that the changes did not significantly affect the induced levels of expression from Ptrc in *S. elongatus*, we performed western blots to detect EngA from extracts of 3^N^Ptrc-EngA or 3^N^Ptrc^Osym^-EngA cultures induced with 1 mM IPTG (Figure 3C). As expected, no significant differences were obtained at the 3 or 24 h timepoints, and thus the *lac* operator mutation does not impair IPTG-induction.

Next, the Δ*engA* allele was transformed in parallel into 3^N^Ptrc-EngA and 3^N^Ptrc*^Osym^*-EngA. Subsequent PCR analysis of chloramphenicol-resistant (Cm^R^) clones confirmed the allelic replacement of the *S. elongatus engA* locus by *cat*, giving strains 3^N^Ptrc-EngA*/*Δ*engA* and 3^N^Ptrc*^Osym^*-EngA*/*Δ*engA* (Figure 3B, lower panels). However, rapid segregation of the inactive allele independently of IPTG also occurred for strain 3^N^Ptrc*^Osym^*-EngA*/*Δ*engA*, indicating that there was still enough promoter leakage from *Ptrc^Osym^*::*engA* to complement EngA essential functions.

Western analysis of protein extracts from strains WT, 3^N^Ptrc-EngA*/*Δ*engA* and 3^N^Ptrc*^Osym^*-EngA*/*Δ*engA* detected small but reproducible differences between them, with both engineered derivatives producing slightly higher levels of EngA (Figure 3D). Importantly, although there was still promoter leakage, repression was more efficient at Ptrc^Osym^ than at Ptrc, in line with the idea that Ptrc can be more efficiently regulated in *S. elongatus* (Geerts et al., 1995) than in *Synechocystis* (Camsund et al., 2014). Thus, by obtaining Ptrc^Osym^ we expanded the repertoire of IPTG-inducible promoters that can be used for gene expression studies in *S. elongatus*.

3^N^Ptrc*^Osym^*-EngA*/*Δ*engA*, expressing at least as much EngA protein as the wild-type strain under standard culture conditions but devoid of its natural regulation, was used alongside other relevant strains to gain further insights into the physiological relevance of EngA upregulation and into the contribution of EngA or the EngA/PipX ratio to growth during high light stress. Information concerning EngA or PipX protein levels, PipX/EngA ratios, and regulation of relevant *engA* alleles of this panel of six *S. elongatus* strains (including the control strains WT and Ptrc) is summarized in Table 3. Note that genes fused to Ptrc are IPTG-inducible but not environmentally controlled.

**Table 3:** Relevant features in standard laboratory culture conditions of the indicated *S. elongatus* strains.

Strain	Relevant allele(s)	Relative protein levels		PipX/EngA Ratio
		PipX	EngA	
WT or 3^N^Ptrc	*engA, pipX*	1	1	1
*ΔpipX*	*engA*	0	1	0
3^N^Ptrc-EngA^a, 2^	*Ptrc::engA, engA, pipX*	1	1.6	0.6
3^N^Ptrc^Osym^-EngA/*ΔengA*^a, b, 3^	*Ptrc^Osym^::engA, pipX*	1	≥1	≤1
1^S^Ptrc-PipX^1^	*Ptrc::pipX, engA, pipX*	4	1	4

^a^Inducible by IPTG, ^b^Non-inducible by environmental stress. Data from ^1^Labella et a., 2017, ^2^Jerez et al.,2021 or ^3^this work (Figure 3D).

### An excess of EngA does not stimulate growth of *S. elongatus* at high light

Because growth measurements of liquid cultures under high light were always very variable, drop-plate assays were most convenient since they allow direct comparison between strain derivatives and their controls on the same Petri dish. It is worth noting that the growth sensitivity of *S. elongatus* to excess light is far greater on solid than on liquid media, and thus the light intensities for experiments on plates under either our standard or “moderate” light conditions (ML, 50 μmol m^−2^ s^−1^) or high light conditions (HL, 400 μmol m^−2^ s^−1^) are lower than those used for the liquid cultures.

**Figure 5:**
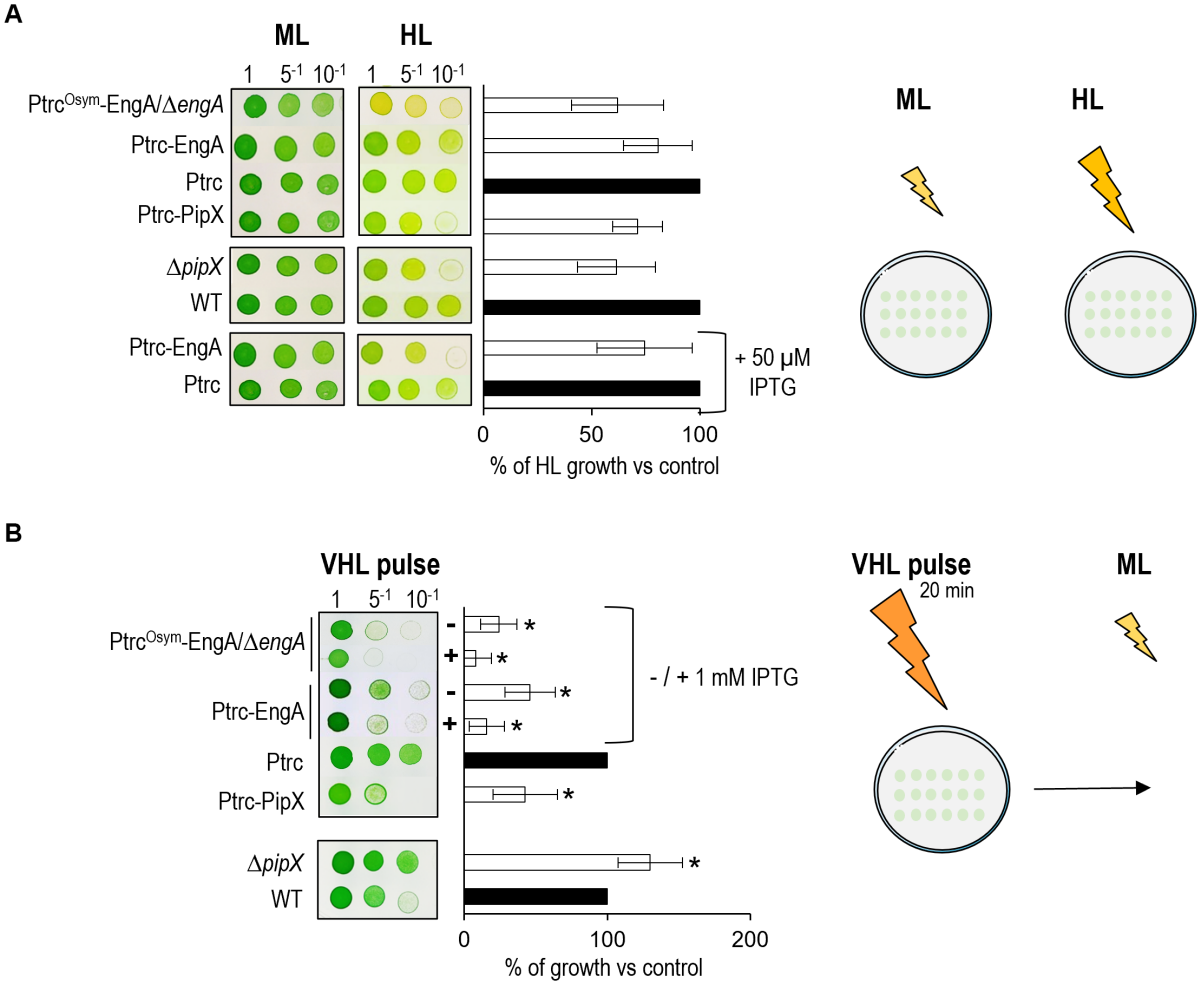
Growth response of *S. elongatus* strains subjected to two different light treatments. Relevant light conditions or treatments are schematically illustrated to the right of each experimental panel. **(A)** Growth tests under moderate or standard (ML) and high light (HL) intensities (50 and 400 μmol m^−2^ s^−1^) and **(B)** after a 20 min pulse of very high light (VHL, 850 μmol m^−2^ s^-1^). Cultures were grown for 24 h until OD_750nm_ of 0.5, diluted, and drop-plated. Where specified, IPTG was added at the indicated concentrations on plates **(A)** or to cultures 2 h before plating **(B)**. Photographs were taken after 5 days. Data are presented as means and error bars (standard deviation) of two to six independent experiments. Statistically significant differences (value of *p* < 0.05) in a pairwise Wilcoxon test with Bonferroni correction are indicated with *.

The results of drop-plate assays for the panel of strains listed in Table 3 are shown in Figure 5A. In high light 3^N^Ptrc*^Osym^*-EngA*/*Δ*engA* grew slower than the 3^N^Ptrc control strain, a result supporting the importance of upregulation of EngA levels for growth in these stress conditions. However, strain 3^N^Ptrc-EngA, either in the absence or in the presence of IPTG, also grew (slightly) slower than the control in high light. Since strain 3^N^Ptrc-EngA maintains the *engA* locus intact and should therefore be capable of upregulating EngA levels, it appears that in this strain the extra levels of EngA provided by the ectopic *Ptrc*::*engA* allele are not stimulating growth in high light but rather slowing it down. Thus, these results suggest that while the relatively small increase in EngA levels produced by upregulation of the *engA* locus may be important for acclimatization and growth in high light stress, additional or unregulated levels of EngA can also interfere with growth under high light conditions.

It is worth noting that high light is the first experimental condition in which increased EngA levels have rather negative effects on *S. elongatus* growth and that IPTG-induced overexpression of EngA has opposite effects on growth under cold or high light stress, two environmentally relevant conditions in which EngA levels are upregulated. The implication is that the molecular mechanisms and details by which EngA mediates acclimatization to stress must differ between the two studied conditions. The negative role of EngA in the context of high light stress suggests that cyanobacterial EngA may have acquired specific functions related to the photosynthetic lifestyle.

### Physiological levels of PipX are required for maximal growth of *S. elongatus* at high light

Interestingly, both Δ*pipX* and 1^S^Ptrc-PipX strains grew worse than their controls in high light (Figure 5A). The finding that either eliminating PipX or increasing its levels some 4-fold slowed down growth at high light indicates that maximal growth on high light conditions requires physiological levels of PipX, supporting the idea that significant alterations of the PipX/EngA ratio have negative impacts on *S. elongatus* growth at high light.

This is, to our knowledge, the first report of an environmental condition in which wild type *S. elongatus* has growth advantage over the Δ*pipX* mutant, implying that PipX plays a positive role specifically under conditions of high light stress. Given the ability of PipX to bind to EngA, presumably to sequester it, it is tempting to propose that, in addition to interference with EngA activity and growth, PipX may also interfere with the EngA-mediated inhibition of growth observed in high light.

### Growth of *S. elongatus* at high light versus growth recovery after a pulse of very high light

High light damages the photosynthesis machinery, leading to photoinhibition of photosystem II (PSII), which is a protein-pigment complex particularly susceptible to photodamage. The repair of photodamaged PSII requires *de novo* synthesis of proteins, particularly of PSII subunit D1, a process which is also sensitive to oxidative stress caused by an accumulation of ROS (Reactive Oxygen Species; Nishiyama et al., 2001, 2004). However, during the acclimatization of *Synechocystis* PCC6803 (hereafter *Synechocystis*) to very strong light, protein synthesis is accelerated, particularly in the case of protein D1 (Jimbo et al., 2019). In *Synechocystis* growth rate was correlated with the rate of synthesis of the D1 protein under different light intensities (Jimbo et al., 2019).

ROS inhibits the synthesis of D1 and of almost all proteins at the elongation step of translation (Nishiyama et al., 2001, 2004) and increases the levels of *psbA* mRNA (encoding D1) that is not associated with ribosomes, suggesting that the initiation of translation may be a target of ROS (Nishiyama et al., 2001). Interestingly, a connection between EngA and the PSII repair cycle has already been reported in *A. thaliana* thylakoids (Kato et al., 2018), suggesting to us that EngA may be regulating translation initiation and, as a result, growth under high light and that the sign of this regulation, positive or negative, would depend on the time that cultures have been exposed to high light. That is, we reasoned that the role of EngA would be different after a drastic increase in light intensity than later on, when multiple acclimatization responses are already operating.

To test this idea with an independent type of drop-plate assay, and hopefully to increase phenotypic differences amongst the studied strains, we next analyzed the growth recovery of *S. elongatus* cultures after a relatively short pulse of very strong light. In particular, a 20′ pulse of 850 μmoles photons m^−2^ s^−1^ was applied to culture drops before the plates were incubated under the moderated light of standard conditions (Figure 5B). When it was pertinent to increase the levels of EngA, IPTG was added to cultures 2 h before drop plating.

### EngA and PipX are both involved in delaying growth recovery after a very intense pulse of high light

Comparison between the two types of experiments shown in Figures 5A,B indicated that the growth recovery assay provided higher sensitivity than the growth assays under continuous high light, strengthening the previously observed negative impact of PipX or EngA excess under continuous high light. In fact, the negative impact of the EngA excess was already observed with non-treated cultures of 3^N^Ptrc-EngA or 3^N^Ptrc*^Osym^*-EngA*/*Δ*engA* and further increased in IPTG-treated cells, thus confirming the relevant role of EngA in promoting high light-dependent growth delay.

Therefore, independent assays confirmed that in addition to its growth-promoting function, EngA can also inhibit growth. Given the evolutionary conservation and complexity of interactions involved in the ribosome-assembly function of EngA, the role of EngA on growth inhibition upon high light exposure would presumably be the result of interfering with translation initiation.

Interestingly, the Δ*pipX* strain recovered faster than the control, indicating that PipX delayed growth recovery after the high light pulse. This result, apparently at odds with the slower growth of Δ*pipX* under continuous high light illustrates that the two types of assays give complementary information in relation to the response to high light stress. While PipX stimulated growth specifically under continuous high light it interfered in a dosage-dependent manner with growth recovery after a high light pulse, suggesting that PipX enhances the inhibitory role of EngA upon sudden high light exposure.

### The distinctive signatures of cyanobacterial EngA proteins include a redox motif

The inferred role of EngA as a sensor of high light stress and its physical interaction with PipX, a cyanobacterial hallmark protein, suggested that cyanobacterial EngA has acquired specific functions related to the photosynthetic lifestyle. If that was the case, these differences may encompass specific structural features distinguishing cyanobacterial EngA proteins from their prokaryotic homologs. With this in mind, we searched for cyanobacterial signatures based on the protein sequence.

**Figure 6:**
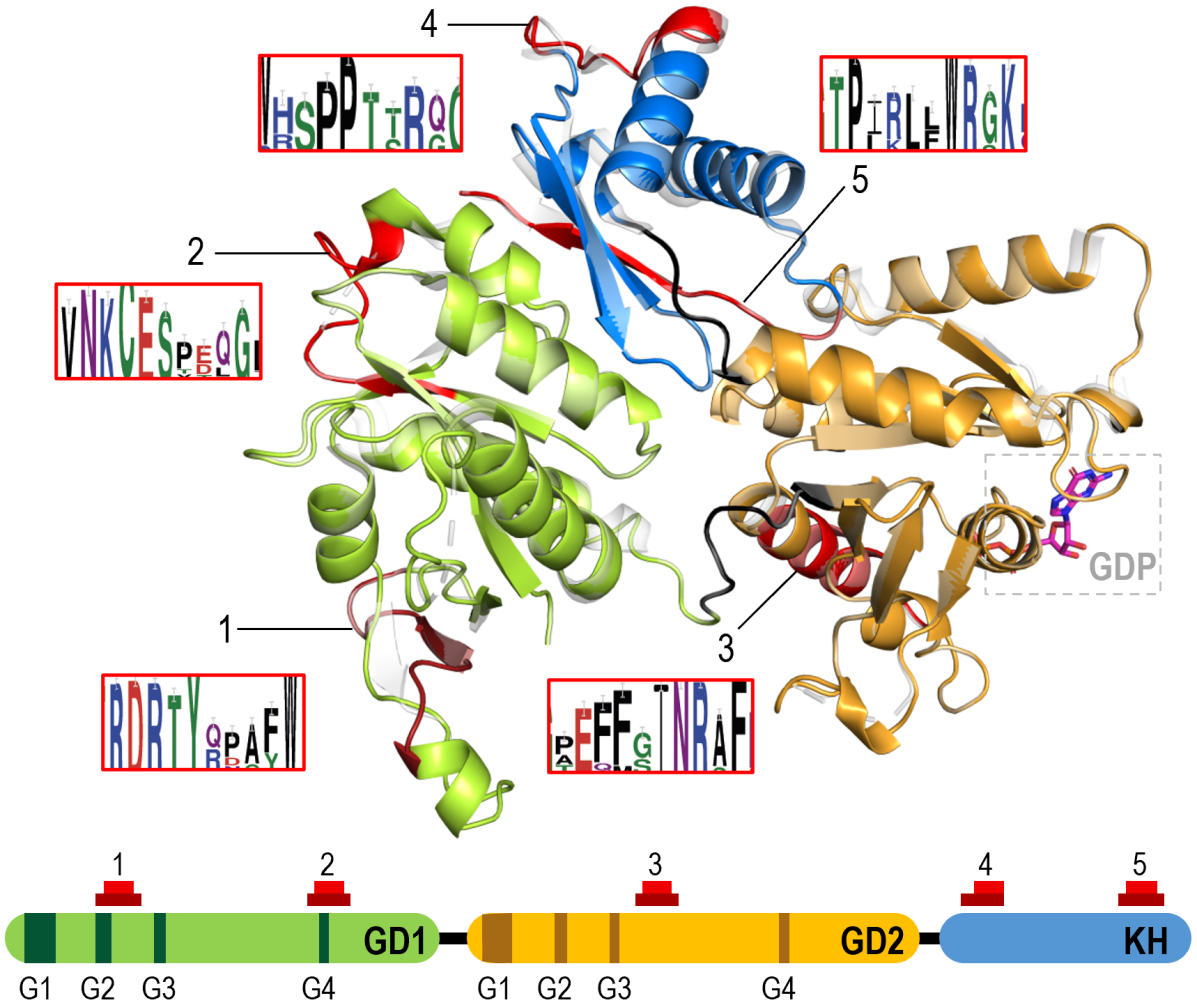
Distinctive features of cyanobacterial EngA proteins. SWISSmodel prediction of EngA structure with GD1, GD2 and KH domains colored green, orange and blue, respectively. A GDP molecule binding to GD2 is also shown. Prediction is based on *Bacillus subtilis* EngA structure, which is superimposed using Pymol and shown in grey. Combined results of two MEME discriminative analyses of cyanobacterial versus non-cyanobacterial EngA sequences are shown and numbered from N- to C-terminal direction. Positions corresponding to the two sets of results (12 and 18 length motifs, red and dark colors, respectively) are shown and EngA weblogos are indicated close to their location in the structure. G1-4 motifs in GD1 and GD2 are indicated as darker bars.

Preliminary searches using ScanProsite and InterProScan with the *Synpcc7942_2340* protein sequence just detected the already well-known motifs of GTPases. To search for specific motifs that may be present exclusively in cyanobacterial EngA proteins, we compared cyanobacterial and non-cyanobacterial EggNOG EngA/COG1160 sequences with MEME discriminative mode (Bailey and Elkan, 1994). The results of two independent searches with a motif maximum length of 12 or 18 amino acids agreed in the top 5 e-value hits, identifying five regions of 12 amino acids significantly overrepresented in cyanobacteria which are distributed alongside the protein sequence (Figure 6; Supplementary Figure S1; Supplementary Table S2).

The first sequence (TRDRTYXXXXWX) overlaps with the GD1-G2 motif and includes two additional highly conserved aromatic residues. The second sequence (AVNKCESXXXGX) expands the GD1-G4 motif. It includes, at the non-conserved (X) position of the canonical NKX[D/E] G4 motif, a cysteine (Cys122 in *S. elongatus*) which is invariant in cyanobacteria and plants, with the notable exception of *Gloeobacter*, a cyanobacterium without thylakoids. The third sequence (GXEFFXINRXFK) is located between motifs GD2-G3 and GD2-G4 and contains a highly conserved submotif (INRXF). The fourth sequence (XWXSPPXXRXGX) is at the beginning of the KH domain and shares two consecutive prolines with the non-cyanobacterial sequences. The fifth sequence (GTPXRLXWRGKX), located close to the C-terminus of the protein is characterized by a highly conserved tryptophan.

The second signature sequence was very interesting since the cysteine, which is a hallmark of the AVNKCESXXXGX cyanobacterial signature, is often found at the X position of the G4 motif of RAS GTPases and subjected to posttranslational modification in the context of redox signaling (Lander et al., 1997; Hobbs et al., 2013; Messina et al., 2019). Since photosynthesis is a major source of redox agents (Hamilton, 2019) and these increase with light intensity, the redox-mediated inactivation of EngA would provide a mechanism to transfer redox information to the translation machinery. It would also explain the inhibitory role of EngA, triggered by exposure to drastic increases in light intensity, on the growth of *S. elongatus* cultures.

In summary, this analysis, showing distinctive structural features characteristic of the cyanobacterial-chloroplast lineage, provides a working hypothesis for the rather complex regulatory role of EngA in the context of high light stress. In particular, the inhibitory role of EngA would be associated with the oxidation of the conserved residue Cys122.

### Cys122 at EngA is not essential in *S. elongatus*

To investigate the *in vivo* importance of Cys122 in cyanobacterial EngA we independently introduced mutations to encode Ala or Ser at the *engA* codon for Cys122 in plasmid pUAGC87. The resulting plasmid derivatives (pUAGC37 and pUAGC38) were then used to deliver *Ptrc^Osym^*::*engA*^C122A^ or *Ptrc^Osym^*::*engA*^C122S^ sequences, respectively, to the *S. elongatus* chromosome (Fig. S2), using the same procedure described above for the parental gene fusion.

Construction of strains 3^N^Ptrc^Osym^-EngA, 3^N^Ptrc^Osym^-EngA^C122A^, and 3^N^Ptrc^Osym^-EngA^C122S^ was carried out in parallel and verified first by PCR to detect the presence of the ectopic alleles from the Nt^R^ transformants, and then by RFLP analyses with *AluI* (C122A) or *Hyp188I* (C122S) to detect restriction sites incorporated with the mutations. Most of the *AluI* and all of the *Hyp188I* analyzed clones carried the corresponding mutation in homozygosis (Fig. S2). Verified clones with the C122A or C122S were named as 3^N^Ptrc^Osym^-EngA^C122A^ or 3^N^Ptrc^Osym^-EngA^C122S^, respectively.

**Figure 7:**
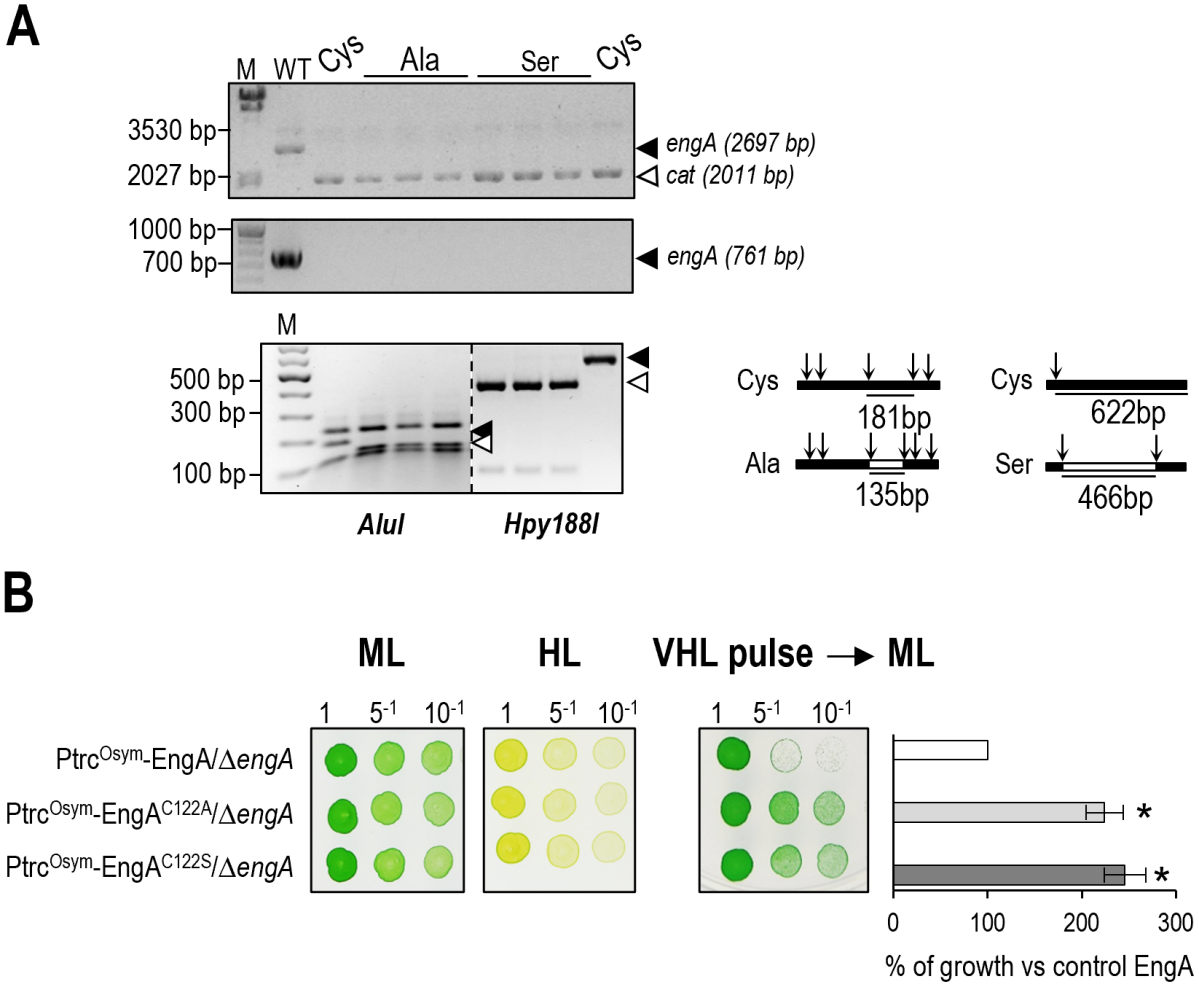
Mutations C122A or C122S at EngA are viable and prevent growth inhibition upon exposure to very intense light. **(A)** PCR analysis (top) showing the complete inactivation of *engA* in strains carrying alleles *Ptrc^OSym^::engA* (Cys, Ala or Ser) and RFLPs (bottom) of the same clones identifying the corresponding point mutations. *engA* digestion patterns and discriminative fragments generated by *AluI* or *Hyp188I* are also specified. Black (WT) or white (mutant) arrowheads point to PCR products or specific restriction bands. **(B)** Growth tests recovery of the indicated strains. Data are presented as means and error bars (standard deviation) of three independent experiments. Other details as in Figure 5.

Strains 3^N^Ptrc^Osym^-EngA^C122A^ and 3^N^Ptrc^Osym^-EngA^C122S^, in parallel with control strain 3^N^Ptrc^Osym^-EngA were then used to inactivate *engA* by allelic replacement exactly as before. PCR confirmed the complete segregation of the Δ*engA* allele in the absence of IPTG and subsequent restriction analysis confirmed that only the point mutant alleles were present in the Cm^R^ selected clones (Figure 7A). Since both 3^N^Ptrc*^Osym^*-EngA*/*Δ*engA* and 3^N^Ptrc*^Osym^*-EngA^C122A^*/*Δ*engA* strains were viable, we concluded that Cys122 is not essential in *S. elongatus*. Furthermore, the growth of strains 3^N^Ptrc*^Osym^*-EngA*/*Δ*engA*, 3^N^Ptrc*^Osym^*-EngA^C122A^*/*Δ*engA,* or 3^N^Ptrc*^Osym^*-EngA*/*Δ*engA*^C122S^ on drop-plate assays under standard conditions (Figure 7B, ML panel) was indistinguishable.

### Substitutions at Cys122 of EngA prevent growth inhibition upon exposure to very intense light

As shown in Figure 7B, growth tests performed under continuous high light did not reveal significant differences between strains 3^N^Ptrc*^Osym^*-EngA*/*Δ*engA*, 3^N^Ptrc*^Osym^*-EngA^C122A^*/*Δ*engA,* or 3^N^Ptrc*^Osym^*-EngA^C122S^*/*Δ*engA*. In contrast, recovery from a pulse of very high light was dramatically faster in the two mutant strains, with no appreciable differences between them, thus indicating that the Cys122 residue of EngA was involved in the growth delay triggered by exposure to the very strong light and that the response was suppressed by the conservative mutations C122A or C122S.

Therefore, the results suggest that the conserved NKCES motif is mediating redox signaling and that Cys122 is required to inhibit growth because of the ability to sense the rapid and dramatic increase in ROS triggered by drastic increases in light intensity. The comparison of the outcomes from the two types of drop-plate assays in the context of high light also illustrates the challenges of gaining information on novel signaling pathways and mechanisms from *in vivo* or genetic approaches, emphasizing the importance of using appropriated or diverse experimental designs.

### Redox regulation of EngA, a new target of ROS in cyanobacteria

The involvement of ribosome-related factors in redox signaling was first reported in the context of translation elongation in *Synechocystis*, where ROS inhibits the synthesis of D1 and of almost all proteins at the elongation step of translation (Nishiyama et al., 2001, 2004). The elongation factors EF-Tu and EF-G are direct targets of ROS, they are inactivated via oxidation of conserved cysteine residues in *Synechocystis* and in the plant model system *A. thaliana* (Ejima et al., 2012; Yutthanasirikul et al., 2016; Jimbo et al., 2018; Toriu et al., 2023). Our results, showing the involvement of Cys122 from EngA in the growth inhibition of *S. elongatus* triggered by high light, add the ribosome-assembly protein EngA to the repertoire of ribosome-related factors transmitting redox signals generated by photosynthesis activity. Therefore, ribosome assembly would also be a key target of ROS in oxygenic photosynthetic organisms, where Cys at the GD1-G4 motif NKCES is a hallmark of EngA.

### Model for redox regulation of EngA and contribution to translation and growth

**Figure 8:**
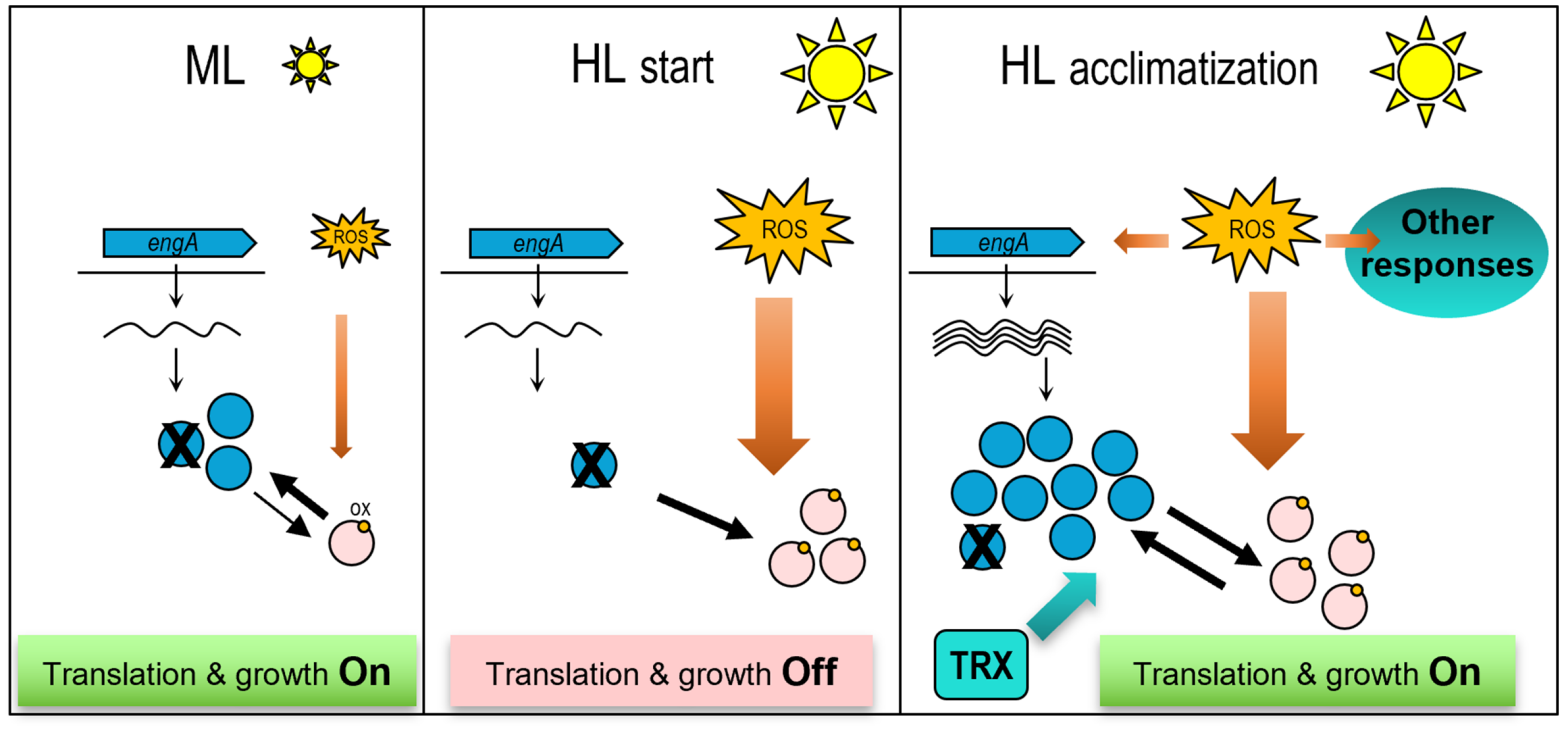
Model for redox regulation of EngA under high light and impact on translation and growth. Left panel: Under moderate light conditions, most EngA molecules are active (reduced), stimulating translation and growth. *Middle panel:* A drastic increase in light intensity (HL) increases ROS leading to massive oxidation of EngA, that becomes inactive and interferes with translation and growth. *Right panel:* The increase in ROS upon the initial exposure to HL triggered multiple protective transcriptional responses, including up regulation of the levels of EngA and activation of thioredoxins. This displaces the redox equilibrium towards reduction of EngA, that in turn is able to sustain translation and growth. A number of PipX-EngA complexes (represented by the X on the EngA molecules) would form in each of the three scenarios, with different outcomes. When EngA is at its basal levels, PipX binding would mainly interfere with EngA functions at the ribosome, but when EngA is abundant and ROS generation important binding to PipX would mainly protect it from EngA inactivation. For simplicity only PipX binding to the active form of EngA has been represented (see text for additional details).

To integrate previous literature with the information gained in this work we propose a model for redox regulation of EngA, summarized in Figure 8.

During moderate light growth conditions, simplified here as ML, EngA is expressed at basal levels. The EngA pool would be mainly in the reduced and active form, so basal levels would be sufficient to promote ribosome assembly, therefore allowing translation and culture growth.

Immediately upon exposure to high light (HL start), ROS would trigger the oxidation of EngA at Cys122, inactivating most of the EngA pool, causing interference with EngA functions in translation initiation and slowing down growth. Whether this inactivation is also accompanied by self-aggregation, as it is the case with EF-Tu or EF-G, cannot be excluded. In this context, it is tempting to speculate that the ability of EngA to self-interact in bacterial two-hybrid assays for protein–protein interactions (Jerez et al., 2021), could reflect a tendency to self-aggregation that could also increase upon oxidation.

The drastic increase in ROS upon exposure to high light would trigger multiple transcriptional and posttranscriptional responses, including upregulation of the levels of ribosome-related factors such as EngA (Figure 2) or EF-Tu (Jimbo et al., 2019) and activation of redox-active enzymes such as thioredoxins (Mallén-Ponce et al., 2022). Together these responses would facilitate subsequent acclimatization to high light.

During long-term exposure to high light (HL acclimatization) and despite the continuous generation of ROS, the enlarged EngA pool would be in an active equilibrium between reduced and active states thanks to the action of thioredoxins. As a result of this and other protective mechanisms, EngA would stimulate translation and growth.

What is the role of PipX in this model? On the one hand, it appears that PipX toxicity, that is, the negative effect of PipX levels on *S. elongatus* growth, is accentuated in high light, conditions in which EngA activity is compromised by oxidative stress and a relatively low EngA/PipX ratio would interfere with EngA functions and growth. This is inferred from the impaired growth of strain 1^S^Ptrc-PipX on high light and its slower recovery after a high light pulse, as well as by the faster recovery of the null *pipX* mutant after a high light pulse. On the other hand, the slightly impaired growth of the *pipX* mutant in high light indicated that during long-term exposure to high light PipX also plays a positive role and here it is tempting to propose that binding of PipX to part of the enlarged EngA pool favors the active form of EngA in detriment of the inactive one. Whether the formation of PipX-EngA complexes prevents oxidation of EngA and/or facilitates reactivation by assisting interactions with thioredoxins are two possibilities worth investigating.

## Data availability statement

The raw data supporting the conclusions of this article will be made available by the authors, without undue reservation.

## Author contributions

AC designed research. AL, SB, RC, and PS performed research. AC wrote the paper. All authors analyzed data, contributed to manuscript revision, read, and approved the submitted version.

## Funding

This work was supported by grant PID220-118816GB-I00 from the Spanish Government (MICINN) and grants VIGROB22-126 and VIGROB23-126 from the University of Alicante. SB is supported by a National Grant from the Algerian Ministry of Higher Education and Scientific Research.

## Conflict of interest

The authors declare that the research was conducted in the absence of any commercial or financial relationships that could be construed as a potential conflict of interest.

## Publisher’s note

All claims expressed in this article are solely those of the authors and do not necessarily represent those of their affiliated organizations, or those of the publisher, the editors and the reviewers. Any product that may be evaluated in this article, or claim that may be made by its manufacturer, is not guaranteed or endorsed by the publisher.

## Footnotes

^1^ http://eggnogapi5.embl.de/nog_data/text/raw_alg/COG1160

^2^ http://eggnogapi5.embl.de/nog_data/text/raw_alg/ENOG5037JM3

^3^ http://weblogo.threeplusone.com/create.cgi
