# Supplementary material for: The ribosome assembly GTPase EngA is involved in redox signaling in cyanobacteria
Source: Front Microbiol. 2023 Aug 10;14:1242616. doi: 10.3389/fmicb.2023.1242616 (PMC10448771; doi:10.3389/fmicb.2023.1242616)
Supplement: Supplementary file 1 [file Presentation_1.PPTX]

## Slide 1
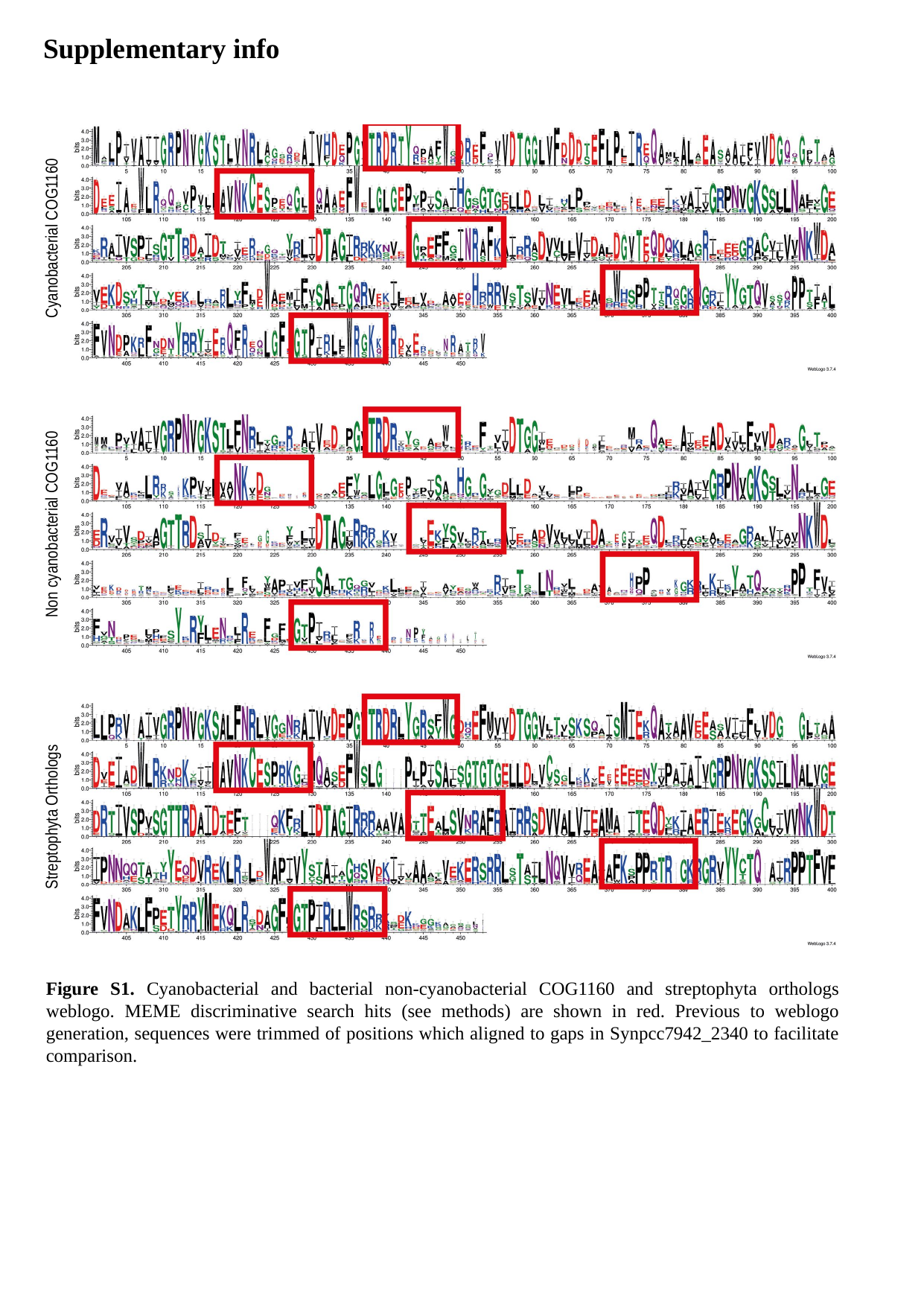

Supplementary info
Cyanobacterial COG1160
Non cyanobacterial COG1160
Streptophyta Orthologs
Figure S1. Cyanobacterial and bacterial non-cyanobacterial COG1160 and streptophyta orthologs weblogo. MEME discriminative search hits (see methods) are shown in red. Previous to weblogo generation, sequences were trimmed of positions which aligned to gaps in Synpcc7942_2340 to facilitate comparison.

## Slide 2
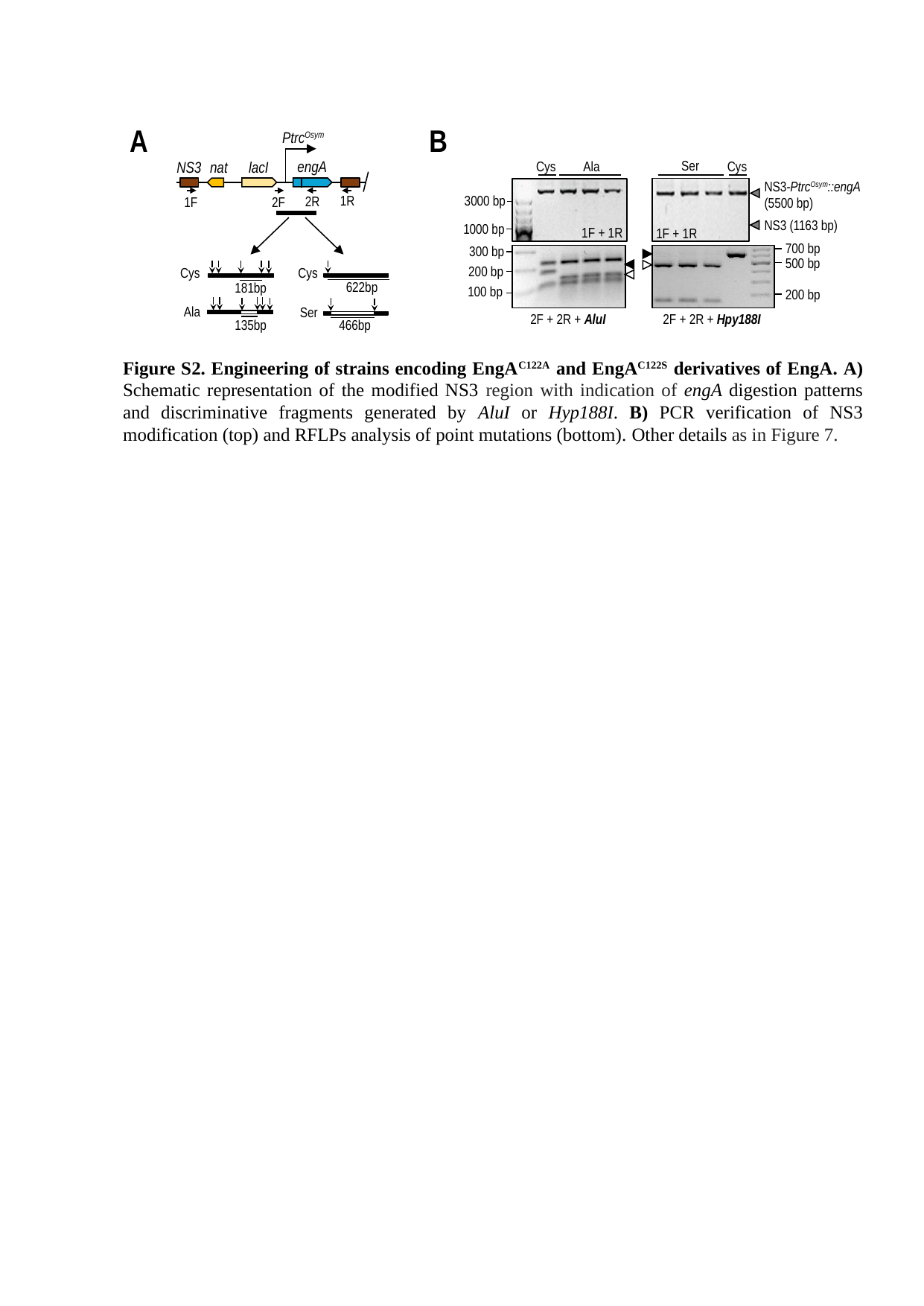

A
B
PtrcOsym
Ser
engA
Cys
Ala
Cys
NS3
nat
lacI
NS3-PtrcOsym::engA (5500 bp)
1R
2R
3000 bp
1F
2F
NS3 (1163 bp)
1000 bp
1F + 1R
1F + 1R
700 bp
300 bp
500 bp
200 bp
Cys
Cys
622bp
181bp
Ala
Ser
135bp
466bp
100 bp
200 bp
2F + 2R + AluI
2F + 2R + Hpy188I
Figure S2. Engineering of strains encoding EngAC122A and EngAC122S derivatives of EngA. A) Schematic representation of the modified NS3 region with indication of engA digestion patterns and discriminative fragments generated by AluI or Hyp188I. B) PCR verification of NS3 modification (top) and RFLPs analysis of point mutations (bottom). Other details as in Figure 7.
