# Supplementary material for: The ribosome assembly GTPase EngA is involved in redox signaling in cyanobacteria
Source: Front Microbiol. 2023 Aug 10;14:1242616. doi: 10.3389/fmicb.2023.1242616 (PMC10448771; doi:10.3389/fmicb.2023.1242616)
Supplement: Supplementary file 2 [file Table_1.DOCX]

**Table S1.** Oligonucleotides used in this work.

| **Primer ID** | **Sequence** | |
| --- | --- | --- |
| Ptrc_symmetric_F | 5' GTGAGCGCTCACAATTTCACACAGGAAACAGACCATGGAATTC 3' |  |
| Ptrc_symmetric_R | 5' GAAATTGTGAGCGCTCACAATTCCACACATTATACGAGCCGG 3' |  |
| Mut_C122A-F | 5' CGTGGTGGCAGTGAACAAAGCTGAATCGCCGGATAAAGGCGC 3' |  |
| Mut_C122A-R | 5' GCGCCTTTATCCGGCGATTCAGCTTTGTTCACTGCCACCACGATC 3' |  |
| EngA-C122S-For | 5' CGTGGTGGCAGTGAACAAATCCGAATCGCCGGATAAAGGCGC 3' |  |
| EngA-C122S-Rev | 5' GCGCCTTTATCCGGCGATTCGGATTTGTTCACTGCCACCACGATC 3' |  |
| NS3-seq-1F | 5' ACCTCCGGCAGTCAATTA 3' |  |
| NS3-seq-1R | 5' AGGGACTGGTTGATCGGT 3' |  |
| PIPX-5R-129 | 5' CAGCCCGCAAATCAGCAG 3' |  |
| PipX-126-F | 5' TAAAAACTAGCCGCCCTTGC 3' |  |
| 2340-For | 5' CCGAGGATCCTGATGTGACTGGCGC 3' |  |
| 2341-rev | 5' CAGAGTCGACGCCATTGACTGAGG 3' |  |
| EngA-seq-4R | 5' GTCCCCAGCTGCTTCATC 3' |  |
| EngA-3R | 5’ CGTTGACAAACAGGGTGAAGCTA 3’ |  |
| Ptrc-2F-seq | 5' GCGCCGACATCATAACGG 3' |  |
