# Supplementary material for: The ribosome assembly GTPase EngA is involved in redox signaling in cyanobacteria
Source: Front Microbiol. 2023 Aug 10;14:1242616. doi: 10.3389/fmicb.2023.1242616 (PMC10448771; doi:10.3389/fmicb.2023.1242616)
Supplement: Supplementary file 3 [file Table_2.DOCX]

**Table S2.** MEME discriminative search results.

| **Motif** | **Max. Length^1^** | **Log Likelihood Ratio** | **E-value** | **Position^2^** | **Motif** |
| --- | --- | --- | --- | --- | --- |
| SWHSPPTTRQGK | 12 | 4204 | 3.8×10^-1246^ | 370 | SWHSPPTTRQGK |
| AVNKCESPEQGL | 12 | 4173 | 4.2×10^-1242^ | 118 | AVNKCESPEQGL |
| GPEFFGINRAFK | 12 | 4517 | 2.7×10^-1386^ | 244 | GPEFFGINRAFK |
| TRDRTYQPAFWG | 12 | 4221 | 9.3×10^-1255^ | 38 | TRDRTYQPAFWG |
| GTPJRLLWRGKK | 12 | 4107 | 1.3×10^-1202^ | 428 | GTPJRLLWRGKK |
| GGLVFDDDTEFL | 12 | 4312 | 9.6×10^-1294^ | 59 | GGLVFDDDTEFL |
| QGRIYYGTQVS | 12 | 4131 | 2.4×10^-1232^ | 382 | QGRIYYGTQVS |
| RLYFLDWAEMIF | 12 | 3960 | 1.2×10^-1136^ | 318 | RLYFLDWAEMIF |
| AGRIEEEGRACV | 12 | 3818 | 1.8×10^-1072^ | 281 | AGRIEEEGRACV |
| KRFNDNYRRYIE | 12 | 4178 | 2.5×10^-1234^ | 406 | KRFNDNYRRYIE |
| VLEEAVSWHSPPTTRQGK | 18 | 5785 | 1.0×10^-1842^ | 364 | VLEEAVSWHSPPTTRQGK |
| PGVTRDRTYQPAFWGDRE | 18 | 6155 | 1.2×10^-2009^ | 35 | PGVTRDRTYQPAFWGDRE |
| VLLAVNKCESPEQGLIQA | 18 | 5666 | 1.1×10^-1792^ | 115 | VLLAVNKCESPEQGLIQA |
| VEYGPEFFGINRAFKAIR | 18 | 6438 | 4.5×10^-2122^ | 241 | VEYGPEFFGINRAFKAIR |
| GFEGTPJRLLWRGKKQRD | 18 | 5753 | 1.6×10^-1829^ | 425 | GFEGTPJRLLWRGKKQRD |
| GGLVFDDDTEFLPLIRZQ | 18 | 6404 | 4.3×10^-2120^ | 59 | GGLVFDDDTEFLPLIRZQ |
| VNDPKRFNDNYRRYIERQ | 18 | 6260 | 1.3×10^-2055^ | 402 | VNDPKRFNDNYRRYIERQ |
| ELRSRLYFLDWAEMIFVS | 18 | 5239 | 5.8×10^-1599^ | 314 | ELRSRLYFLDWAEMIFVS |
| AGRIEEEGRACVIVVNKW | 18 | 6140 | 6.6×10^-2003^ | 281 | AGRIEEEGRACVIVVNKW |
| LGEPYPISAIHGSGTGEL | 18 | 6355 | 1.5×10^-2099^ | 140 | LGEPYPISAIHGSGTGEL |

^1^Maximum length allowed in the search.

^2^Starting position of the motif in *S. elongatus* homolog protein sequence.
